# Exercise Interventions and Pregnancy-Related Back Pain: Evidence and Gaps From a Systematic Review

**DOI:** 10.3389/ijph.2026.1608730

**Published:** 2026-04-09

**Authors:** Jinchen Chen, Hongli Yu, Dagmara Damps, Anna Szumilewicz

**Affiliations:** 1 Faculty of Physical Culture, Gdansk University of Physical Education and Sport, Gdansk, Poland; 2 The College of Physical Education, Sichuan University of Science and Engineering, Zigong, Sichuan, China

**Keywords:** back pain, exercise, high-intensity interval training, pregnancy, systematic review

## Abstract

**Objectives:**

To review and synthesize existing evidence on exercise interventions of varying intensity for pregnancy-related back pain and to identify directions for future research.

**Methods:**

A systematic review of nine studies involving 1,438 participants was conducted. Interventions focused predominantly on low-to moderate-intensity exercise, and no study employed high-intensity interval training (HIIT). Exercise types included aerobic and resistance training as well as aquatic and stability exercises.

**Results:**

Most studies demonstrated significant reductions in back pain, but methodological heterogeneity and limited sample sizes prevented meta-analytical synthesis. Randomized controlled trials showed moderate risk of bias due to challenges in blinding and randomization, while non-randomized trials had substantial limitations, including inadequate measurement validation and increased risk of bias.

**Conclusion:**

Existing evidence supports the potential benefits of low-to moderate-intensity exercise for pregnancy-related back pain but remains methodologically weak. High-quality studies are needed to evaluate HIIT and other innovative strategies for managing pain and improving maternal health and wellbeing.

**Trial Registration:**

identifier CRD42024578089.

## Introduction

Pregnancy is a significant phase in a woman’s life. During this stage, pregnant women experience physiological and psychological alterations, and hormonal variations and uterine expansion (along with the resulting shift in center of gravity) may induce physiological and biomechanical modifications that increase susceptibility to pain [1]. Pregnancy-related back pain is a prevalent health concern among women. Studies indicate that approximately 50%–70% of pregnant women experience back pain during pregnancy [2, 3], with severity generally increasing as pregnancy advances [4]. This not only influences sleep and quality of life but may also damage mental health during pregnancy [5]. Consequently, managing pregnancy-related back pain is crucial for enhancing the quality of life and wellbeing of pregnant women.

Previous studies on the management of pregnancy-related back pain have explored various strategies. Among them, acupuncture has shown significant benefits in randomized controlled trials for relieving pelvic and back pain during pregnancy [6, 7], other approaches include Kinesio taping, a drug-free elastic cotton tape used to support musculoskeletal function [8], transcutaneous electrical nerve stimulation (TENS), and osteopathic manipulative treatment [9]. More recently, innovative techniques such as virtual reality (VR) integrated with physical therapy have been proposed as complementary tools to improve outcomes [10]. These emerging technologies may offer an alternative to traditional interventions. While these treatments have demonstrated some efficacy in alleviating pregnancy-related back pain, their implementation is intricate and challenging. Conversely, exercise interventions are extensively promoted because of their simplicity, minimal equipment requirements, and absence of pharmacological side effects. Furthermore, suitable exercise can prevent and mitigate musculoskeletal pain while reducing the related risk of impairment [11]. More studies were conducted to assess the effectiveness of exercise in reducing back pain; however, interventions differed substantially in type, frequency, duration, intensity, and training methods, increasing the diversity of available data.

Recent studies have begun to explore the role of HIIT during pregnancy [12], and previous research indicates that 8-week HIIT positively influences physiological indicators [13], supports the maintenance of normotension in pregnant women [14], significantly improves mental health without inducing negative stress responses [15], and improves pain tolerance in pregnant women [16]. Those findings support considering HIIT as an exercise option beneficial for pregnancy outcomes. Although some studies suggest potential physiological and psychological benefits, its effects on pregnancy-related back pain remain unclear.

There is insufficient research on how varying exercise intensities (low, medium, and high) alleviate pregnancy-related back pain, and the mechanisms underlying pain reduction have not been comprehensively analyzed. Although certain studies have investigated various exercise types for alleviating pregnancy-related back pain, the findings are inconsistent, and the characteristics and efficacy of these interventions, especially for back pain during pregnancy, have not been adequately or systematically examined [17]. Therefore, this research aimed, first, to characterize the components of exercise interventions and analyze their impact on alleviating pregnancy-related back pain through a systematic review and, second, to explore data on the potential effectiveness and feasibility of high-intensity exercise, particularly HIIT, in alleviating back pain during pregnancy.

## Methods

### Protocol and Registration

This systematic review was conducted in accordance with the Preferred Reporting Items for Systematic Reviews and Meta-Analyses (PRISMA) statement [18] and registered with PROSPERO on 09 August 2024 (CRD42024578089). The review methodology is available at https://www.crd.york.ac.uk/PROSPERO/view/CRD42024578089.

### Search Strategy

A systematic literature search was conducted in PubMed and Web of Science between March and May 2024. The same search string was applied to both databases using the following keywords connected by Boolean operators (AND/OR): (“back pain” OR Entry Terms) AND (“pregnancy” OR Entry Terms) AND (“exercise” OR Entry Terms) AND (“high-intensity interval training” OR Entry Terms) AND (“athletes” OR Entry Terms).

No publication year restrictions were applied, and only peer-reviewed articles published in English were considered. This approach ensured reproducibility and transparency.

### Eligibility Criteria

In accordance with Cochrane recommendations, researchers incorporated papers utilizing the PICOS framework (population, intervention, comparison, outcomes, and study design) [19]. The PICOS process is a structured retrieval technique rooted in evidence-based medicine (EBM) that eliminates irrelevant material [20]. Initially, independent researchers examined abstracts for potentially significant information and subsequently employed the PICOS framework. A second stage was utilized to obtain full texts corresponding to abstracts that met the eligibility criteria and potentially relevant publications: full texts linked to eligible abstracts and articles from reference lists were identified. The final stage was a comprehensive evaluation of entire articles for potential inclusion, with the articles assessed for eligibility. When two investigators disagreed on the inclusion of an item in the research, the senior author completed an independent assessment to ascertain the article’s suitability for inclusion. The included criteria are as follows:

1. Pregnant participants regardless of age or stage of pregnancy;
2. The interventions were conducted in participants with normal course of pregnancy, without any health complications (apart from back pain);
3. Exercise interventions, unrestricted by type, frequency, duration or intensity;
4. All trials comprised an intervention group and a control group, featuring various exercise interventions compared to a control group, typically involving standard care or no-exercise conditions;
5. The main outcomes concentrated on low back pain, with no limitations on the measurement scale or the assessment of pain severity;
6. Prospective, experimental studies, including randomized controlled trails (RCTs) and non-randomized controlled trails NRCTs.

The excluded criteria as below:

1. Literature published in non-English languages;
2. The participants presented symptoms of any health problems or pregnancy complications, other than low back pain;
3. Review articles or conference proceedings;
4. Articles without accessible data;

### Extract Data From the Selected Articles

Data was extracted using a standardized form, in compliance with the Cochrane criteria for data extraction in systematic reviews [21]. Two experts independently extracted the data. In cases of discrepancies, a third expert was consulted to reach a consensus. The extracted data included author, publication year, country of study, study population (i.e., number of participants per group), age range (mean and standard deviation), gestational period, outcome measures (back pain), effectiveness of exercise, intervention exercise type, frequency, duration of a single session, intensity, total program duration, and mode of delivery (supervised or unsupervised).

### Methodological Quality and Risk of Bias Assessment

This investigation included both RCTs and NRCTs; two experts independently evaluated bias and methodological quality using the Cochrane and COSMIN tools, respectively. Any discrepancies between the two reviewers were resolved through discussion with a third reviewer, who is also a co-author of this manuscript.

For RCT, risk of bias in the included randomized controlled trials (RCTs) was assessed using the Cochrane Risk of Bias 2.0 (RoB 2) tool, following the guidelines of the Cochrane Handbook for Systematic Reviews of Interventions [22]. The assessment included five key domains: (1) bias arising from the randomization process, (2) bias due to deviations from intended interventions, (3) bias due to missing outcome data, (4) bias in measurement of the outcome, (5) bias in selection of the reported result. Each domain was assessed as “Low risk,” “Some concerns,” or “High risk.” To visualize the risk of bias assessment results, the Robvis package in R was used to generate traffic light plots and summary plots, providing an overview of the distribution of bias risks across the included studies [23].

The COSMIN risk of bias tool should be utilized to evaluate potential bias and methodological quality in NRCTs. This tool offers explicit Cochrane handbook for systematic reviews of interventions criteria to assess a study’s reliability. Each criterion must be assessed independently using the worst-score-count approach to estimate overall risk of bias (irrelevant standards are omitted from the final assessment). The phrase “risk of bias” refers to a methodology used in systematic reviews to evaluate the precision of diagnostic tests and procedures, as specified by Cochrane. The ten criteria of the COSMIN framework encompass critical elements of patient-reported outcome measure (PROM) development, such as content validity, structural validity, internal consistency, cross-cultural validity/measurement invariance, reliability, measurement error, criterion validity, hypothesis testing for construct validity, and responsiveness. These components are assessed using a four-point scale: very good, adequate, dubious, or inadequate. The designation “NA,” representing “not applicable,” may pose difficulties for specific criteria. If a study on structural validity relies on classical test theory (CTT), the criteria for item response theory (IRT) would be irrelevant and should be omitted from the “worst score counts” evaluation. Cells in this standard with a gray background are prohibited for use [24].

## Results

### Included Articles and Their Characteristics

**FIGURE 1:**
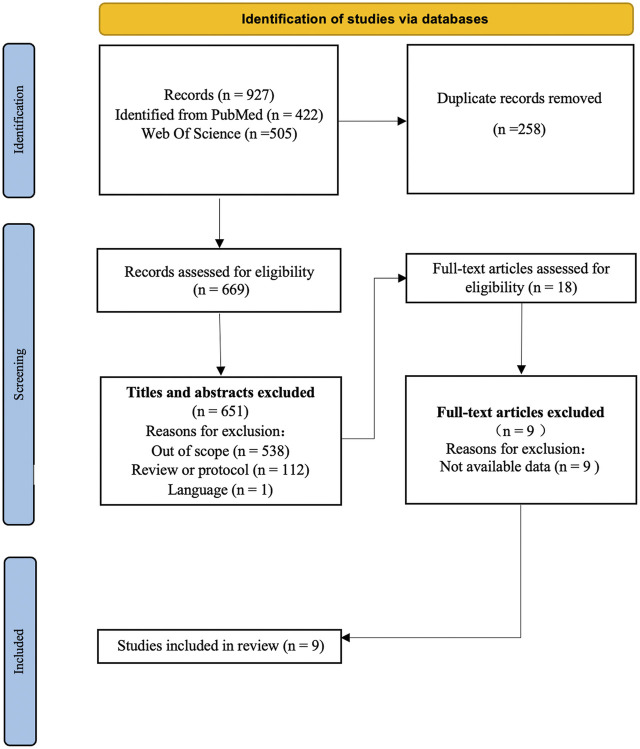
Flow diagram of the study selection process and results based on Preferred Reporting Items for Systematic Reviews and Meta-Analyses (International. 1999–2023).

A total of 927 publications were identified, including 422 from PubMed and 505 from Web of Science. After removal of 258 duplicates, 669 studies remained. Subsequently, 538 out-of-scope studies, 112 reviews or protocols, and 1 non-English language article were excluded. Full texts were reviewed, and 9 articles with unavailable data were excluded. In total, 9 studies were included in the review, with 729 participants in the experimental group and 709 in the control group. Figure 1 illustrates the exclusion process and rationale.

The demographic and intervention characteristics are presented in Tables 1, 2. In the research analyzed, 67% of the papers originated from Europe, 22% from Asia, and 11% from Africa, with publication years spanning 1999 to 2023. Sample sizes ranged from 46 to 470 participants, totaling 1,438 individuals predominantly aged between 20 and 40 years, while the gestational weeks examined spanned 13–24 weeks; most participants exhibited symptoms of back pain, each session lasted 12–60 min, the entire program often spanned 10–12 weeks, and the primary outcome measure was back pain.

**TABLE 1:** Characteristics of the nine reviewed studies (Europe, Asia and Africa. 1999–2023).

Study (year)	Country	Simple size (N; I^1^/C^2^)	Age (mean ± SD^3^)	Gestation trimester	Main outcome	Control group	Effects of the interventions
Aparicio [25]	Spain	N = 93 (49/44)	33 ± 5	16 weeks	LBP^4^	Usual activities	↓ Back pain (significant)
Watelain [26]	France	N = 90 (45/45)	25–35	24 weeks	Pain intensity	Standard care	↓ Back pain (significant)
Backhausen [27]	Denmark	N = 470 (240/230)	31 ± 4	20 weeks	LBP	Standard care	↓ Back pain (significant)
Haakstad [14]	Norway	N = 84 (42/42)	31 ± 4.0	18 ± 4 weeks	PGP^5^ and LBP	Standard care	↓ Back pain (not significant)
Yan [28]	Taiwan China	N = 89 (44/45)	30 ± 3	22 weeks	LBP	Standard care	↓ Back pain (significant)
Kluge [29]	South African	N = 46 (24/22)	20–40	16 weeks	Pain intensity	Standard care	↓ Back pain (significant)
Shim [30]	Korea	N = 56 (29/27)	20–35	17 weeks	LBP	Standard care	↓ Back pain (significant)
Granath [5]	Sweden	N = 266 (132/134)	18–40	16 weeks	LBP and sick leave	Standard care	↓ Back pain (significant)
Kihlstrand [31]	Sweden	N = 244 (124/120)	≥18	18 weeks	LBP and sick leave	Standard care	↓ Back pain (significant)

I - *intervention group* ^*1*^ *; C - control group* ^*2*^ *; SD - standard deviation* ^*3*^ *; LBP- low back pain* ^*4*^ *; PGP - Pelvic Girdle Pain* ^*5*^ *;* ↓ - reduced back pain.

**TABLE 2:** Characteristics of the exercise interventions in the nine reviewed studies (Europe, Asia and Africa. 1999–2023).

Study (year)	Frequency (Sessions/week)	Intensity	Intervention duration	Session duration (min^1^)	Exercise type	Delivery mode
Aparicio [25]	3	Moderate-to-vigorous	∼23 weeks (From 17th to delivery)	60	Concurrent - training	Supervised
Watelain [26]	2	12–14 on B.r.s.^2^	12 weeks (From 24th to 36th)	60	Flexibility, balance, and strength training	Supervised
Backhausen [27]	2	11–15 on B.r.s.	∼13 weeks (16th/17th to 28th/29th week)	45	Water exercise	Unsupervised
Haakstad [14]	2–3	12–14 on B.r.s.	19 weeks (17th to 36th week)	60	Aerobic dance with strength training	Supervised
Yan [28]	≥3	NA^3^	∼20 weeks (From 20th to delivery)	25–30	Stability ball exercise	Unsupervised
Kluge [29]	1	NA	∼10–18 weeks (16th/24th to 26th/34th week)	30–45	Muscle strengthening	Supervised
Shim [30]	5–7	NA	12 weeks (16th-24th to 28th-36th week)	12	Strengthening exercises and pelvic floor exercises	Unsupervised
Granath [5]	1	NA	∼24 weeks (Mid-pregnancy to delivery)	60	Water aerobics	N/A
Kihlstrand [31]	1	NA	∼22 weeks (18th week to delivery)	60	Water-gymnastics	Unsupervised

Min - minutes^1^; B.r.s. - Borg Rating of Perceived Exertion Scale^2^; N/A - Not applicable (not reported)^3^.

### The Characteristics of Exercise Interventions

The evaluated studies analyzed exercise interventions in pregnancy focusing on back pain, which differed significantly in type, frequency, duration, and intensity (Table 2). Exercise types included aerobic, resistance, water, core stability, balance, flexibility, and stability ball exercises. Half of the regimens consisted of 2–3 weekly sessions lasting 25–60 min, with intervention periods spanning from the 16th week until delivery. In four studies, exercise intensity was specified as moderate or moderate-to-vigorous. In five studies, intensity was not specified, but the exercise types suggested moderate or moderate-to-vigorous intensity. No information on high-intensity exercise was found in any study. Among the nine studies, four interventions were supervised, four were unsupervised, and one did not report this information.

### The Efficacy of Exercise Interventions in Alleviating Pregnancy-Related Back Pain

Of the nine studies included, eight demonstrated a statistically significant reduction in back pain in the intervention group compared to controls, whereas one showed no statistically significant reduction (Table 1). Although statistically significant reductions in pain scores were observed in multiple studies, their therapeutic relevance should be interpreted with caution. The Visual Analog Scale (VAS) indicates that a decrease of 1.5–2.0 points is commonly accepted as the Minimally Clinically Important Difference (MCID), signifying the minimal change considered advantageous by patients [32]. Studies like Kihlstrand et al. [31] documented pain reductions surpassing this threshold (e.g., a decrease of 3.5 points), implying a significant clinical effect. Nonetheless, some included trials demonstrated moderate gains that, although statistically significant, may not have achieved a substantially noticeable change in pregnant individuals. The most frequently used instruments included the Visual Analog Scale (VAS), the Oswestry Disability Index (ODI), and the Roland-Morris Disability Questionnaire, while other tools included the Brief Pain Inventory, Low Back Pain Rating Scale, and study-specific structured questionnaires or interviews.

### Quality Assessment

#### RCT Bias Risk Assessment

**FIGURE 2:**
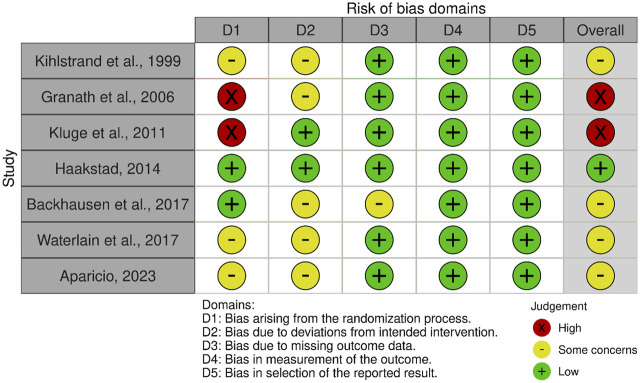
Summary of risk of bias in the randomized controlled trials (Europe and Asia. 2006–2023).

**FIGURE 3:**
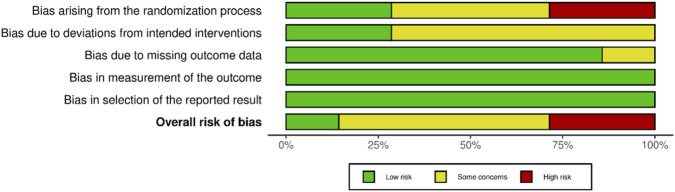
Domain-specific risk of bias across the randomized controlled trials included in the review (Europe and Asia. 2006–2023).

This study utilized the RoB 2 tool to evaluate risk of bias in the included RCTs, with results visualized using the Robvis package. Of the included studies, two had a high risk of bias, while three raised concerns primarily because of issues in the randomization process. Five studies had some concerns regarding deviations from the intended intervention, with three showing low risk in this domain. For missing outcome data, one study was flagged with some concern, while eight exhibited low risk. All studies demonstrated low risk in both outcome measurement and selection of reported results. In the overall assessment, one study exhibited low risk, six raised some concerns, and two had a high risk of bias. Figures 2, 3 illustrate the risk-bias details.

#### Non-Randomized Controlled Trial Bias Risk Assessment

**FIGURE 4:**
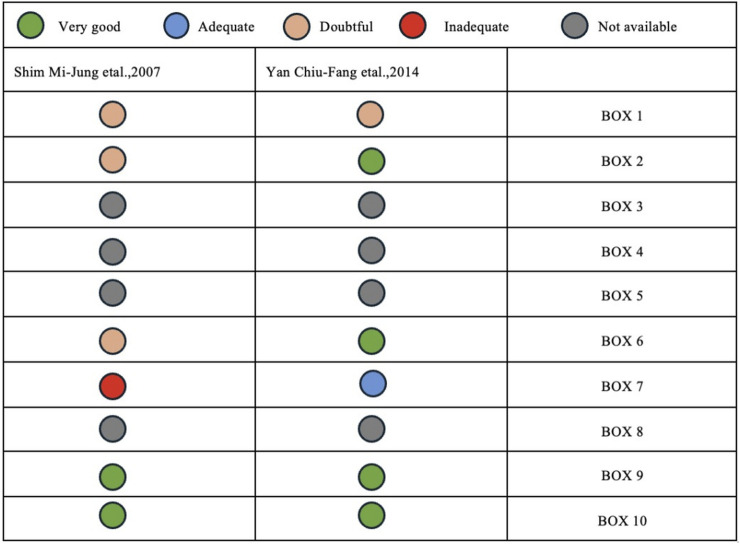
Assessment of study quality and risk of bias in the two non-randomized trials using the Consensus-based Standards for the Selection of Health Measurement Instruments checklist (Europe. 2007–2023). Boxes 1 to 10 represent patient-reported outcome measure development; content validity; structural validity; internal consistency; cross-cultural validity and measurement invariance; reliability; measurement error; criterion validity; hypotheses testing for construct validity; and responsiveness.

Among the two NRCTs considered, COSMINs evaluation of hypothesis testing for construct validity and responsiveness received a rating of “very good.” Nonetheless, structural validity, internal consistency, cross-cultural validity, measurement invariance, and criterion validity were “not available.” Furthermore, one paper was assessed as “inadequate” in reliability and measurement inaccuracy (Figure 4).

## Discussion

The findings of this review suggest that exercise interventions can help prevent and reduce pregnancy-related back pain, with eight of the nine included studies reporting a significant reduction in back pain symptoms following intervention and one showing a non-significant improvement. The interventions fell into three categories: aerobic training, strength training, and mixed methods, including flexibility, balance, and pelvic floor exercises. In four studies, exercise intensity was reported as moderate or moderate-to-vigorous, although the effectiveness of each intervention type varied across studies. None of the included studies evaluated high-intensity exercise protocols, such as HIIT, in this population. This gap highlights a direction for future research, as the potential impact of high-intensity modalities on pregnancy-related back pain remains unexplored. This review primarily maps existing low-to-moderate intensity exercise interventions rather than comparatively analyzing efficacy across intensity levels, and future experimental studies should determine whether exercises of different intensities relieve or prevent back pain during pregnancy.

### The Characteristics of Included Studies

Pregnancy-related back pain is common and often worsens with gestational progression, affecting daily function and quality of life [33]. Addressing it requires biomechanical insight and targeted exercise strategies. Notably, 67% of the studies originated in Europe, likely because of earlier establishment of prenatal exercise guidelines and stronger academic interest, whereas countries like China and South Korea face cultural and institutional barriers to prenatal physical activity promotion [30, 34]. Future research n Asia and Africa is expected to grow with localized approaches. Most studies were published within the past decade, underscoring increased academic focus. In the analyzed studies, low back pain was the predominant outcome, while some extended analysis to pelvic pain or sick leave, reflecting the multifaceted benefits of prenatal exercise on musculoskeletal function.

Exercise interventions varied considerably in type, frequency, duration, and intensity, with early literature revealing substantial inconsistencies in duration and frequency. For example, one intervention lasted 22 weeks with a frequency of once a week [31] and another, 12 weeks with a frequency of 5–7 times weekly [30] Approximately 56% of studies implemented interventions 2–3 times per week, which may constitute an appropriate frequency by offering adequate stimulation for neuromuscular adaptation and strength enhancement while mitigating the dangers of overexertion and attrition prevalent in exercise programs for pregnant individuals [25]. Session lengths typically lasted 30–60 min, and intervention durations ranged from 12 to 36 weeks. All analyzed interventions began in the second trimester, between 16 and 24 weeks of gestation. There are still unfounded concerns that first trimester exercise may be associated with an increased risk of miscarriage [35]. Recent evidence indicates that exercise does not elevate this risk [36]. However, there is still a lack of evidence regarding the outcomes of exercise in early pregnancy, indicating potential for progress in this field [37]. Subsequent research should investigate optimal durations and frequencies of exercise, with particular emphasis on the effectiveness and safety of exercise interventions in early pregnancy. Earlier interventions may be more effective in preventing back pain and early pregnancy-induced abnormalities in posture and biomechanics of movement. Only 4 interventions in the analyzed studies were supervised, which may have affected participants’ health. Supervised exercise, usually conducted by a certified instructor or physiotherapist, enhances adherence through structured accountability and guarantees proper exercise technique [29]. This is crucial for safety and optimizing therapeutic efficacy, particularly for exercises such as targeted core activations.

The analyzed studies included various exercises: aerobic resistance, core, strength, stability, aquatic, and combined training, with aquatic exercises minimizing lumbar pressure and injury risk through buoyancy and low impact, making them particularly beneficial for pregnant women [5, 31]. Studies on stability training, including the use of stability ball, underscored the significance of strengthening pelvic floor muscles to alleviate lower back pain [28, 29]. The reviewed studies corroborated the efficacy of core exercises in enhancing physical fitness and mitigating back pain during pregnancy, highlighting their impact on posture correction [26]. The better effectiveness of specific exercise modalities, such as aquatic training and core stability exercises, may be due to different biomechanical and physiological adaptations. The buoyancy of water markedly alleviates gravitational stress on the spine and pelvic joints, which is especially advantageous during pregnancy when body weight increases, and may promote pain-free mobility and enhance workout capacity [31]. In contrast, core stability exercises engage the deep trunk muscles, such as the transverse abdominis and multifidus. Strengthening this muscle “corset” improves lumbopelvic stability and control, helping mitigate the heightened lumbar lordosis and anterior load of the expanding uterus [29]. Despite the well-recognized benefits of core training in various populations, research on its efficacy for pregnant women remains limited. Future studies should explore diverse exercises, including those effective in non-pregnant populations, and holistic approaches integrating mental health support to strengthen evidence for managing pregnancy-related back pain.

This review also examined whether high-intensity exercise may help alleviate pregnancy-related back pain. However, the included studies did not answer this question. Four interventions involved moderate or moderate-to-vigorous exercise. In three studies, Borg scale values ranged from 11 to 15, suggesting a broad intensity range. In five studies, exercise intensity was not clearly defined, although the interventions were likely of moderate or moderate-to-vigorous intensity. One study suggested that intensity alone may not relieve pregnancy-related lower back pain [17]. Exercise effectiveness likely depends on multiple training components, including duration and type. Two studies used mixed-intensity exercise [25]. This intervention of mixed-intensity training has been useful in alleviating many pregnant symptoms [38], and is regarded as a novel approach to the investigation of pregnancy-related back pain [34]. No included study examined high-intensity exercise or HIIT for pregnancy-related back pain. However, previous studies suggest that HIIT may reduce disability in chronic low back pain and relieve back pain in non-pregnant populations [39]. Exercise physiology studies suggest that HIIT may modulate pain through muscular adaptations and molecular mechanisms, including endogenous opioid release and anti-inflammatory pathway upregulation (e.g., PGC-1α) [40]. The iimplementation during pregnancy has shown beneficial impacts on enhancing cardiovascular function aerobic capacity, and body composition [13], normalizing blood pressure [14] and also on self-assessment of mental health [15]. HIIT may modulate pain through muscular adaptations and molecular mechanisms, including endogenous opioid release and anti-inflammatory pathway upregulation [41], potentially integrating intermittent fetal heart rate monitoring during the later trimesters [42]. Moreover, Future studies should clearly define contraindications and obtain informed consent detailing the risks and key features of high-intensity protocols in this population. HIIT may then be investigated for preventing or alleviating pregnancy-related back pain.

This review covers literature published between 1999 and 2023. In 1985, the American College of Obstetricians and Gynecologists published its first guidelines on exercise during pregnancy [43]. Consequently, advanced Western nations, including Australia [44], Canada (SOGC/CSEP) [45] and the United Kingdom (RCOG) [46], promulgated their own guidelines for exercise during pregnancy, suggesting global acknowledgment and emphasis on the importance of physical activity during this period. Notably, since 2015, the publication of pregnancy physical activity guidelines has significantly increased worldwide. Among 30 guidelines analyzed by the international group of researchers [47], most guidelines (25/30) recommend moderate-intensity exercise during pregnancy, and some cautiously allow high-intensity exercise under professional supervision, although most do not provide tailored advice for highly active women or trimester-specific needs. For example, the guidelines from Spain [48], Australia [47], and Brazil [49] explicitly support the potential feasibility of high-intensity exercise during pregnancy. Spain was the first European country to recommend high-intensity exercise during pregnancy in 2015 [34]. In 2024, Poland updated its guidelines to recommend supervised HIIT for pregnant women under specific conditions [50]. Globally, only four countries—Spain, Poland, Brazil, and Australia—mention high-intensity exercise in pregnancy guidelines. This may partly explain Europe’s stronger focus on pregnancy exercise research, and Poland’s latest guidelines further support HIIT under specific conditions [50]. Further research should examine HIIT for pregnancy-related back pain.

To support interpretation of the review findings, we assessed the quality of the nine included studies. Risk of bias varied across the seven RCTs, mainly due to flaws in randomization, such as inadequate allocation concealment or random sequence generation [5, 29]. Ethical constraints, individualized interventions, and continuous safety monitoring make strict randomization and full blinding impractical in pregnancy-related trials, especially for exercise interventions where participants and instructors are aware of the assigned intervention [51]. Tailoring interventions to maternal health and gestational stage can complicate allocation concealment, increase selection bias, and reduce study reliability; to mitigate this and uncertainty in randomization and intervention delivery, trials may use stratified or adaptive randomization, protocol transparency, blinded outcome assessment, and standardized methods such as centralized randomization and stratified allocation [52]. Study heterogeneity could not be quantitatively assessed, and differences in intervention type, intensity, duration, and outcome measures limited interpretability and generalizability. Future research should therefore adopt more standardized methods and integrate meta-analytic techniques to strengthen the evidence base.

Limitations in Patient-Reported Outcome Measures (PROMs), especially regarding validity and cultural adaptation, may introduce reporting bias, and poor adherence monitoring, randomization, and blinding may compromise intervention integrity; these methodological flaws are important for interpreting study quality and the observed outcomes. Future research should use validated PROMs and objective assessments to improve reliability, while improved trial design or new, specific standards for reporting randomized controlled trials in exercise science are needed to address poor adherence monitoring and inadequate blinding. Most studies showed low bias in missing data, outcome measurement, and result reporting, though adherence and blinding remained crucial for reducing bias in pregnancy trials.

This study used the COSMIN method to assess bias in two NRCTs, revealing key limitations. BOX3, 4, 5, and 8 were “not applicable,” while one paper in BOX7 was rated “inadequate.” Non-randomized trials face inherent challenges: limited resources, small samples, and specific aims, that hinder randomization and blinding, increasing bias risk. Additionally, weak structural validity, unreliable measurements, and lack of cross-cultural validation further undermine credibility and generalizability. Future research should enhance sample diversity and external validity through multicenter designs [53]; employ propensity score matching (PSM) to rectify baseline discrepancies [54]; enhance the standardization of intervention measures [55]; rigorously regulate external variables to improve result reliability [56]; utilize validated instruments and bolster the reliability of data collection [57]. In the data analysis phase, it is crucial to address confounding issues [58], and to prioritize transparent and thorough reporting [55]. Moreover, extended follow-up enhances the longitudinal validity of research [59]. The reliability and validity of NRCTs can be substantially improved by these enhancements.

### Strengths and Limitations

A clear and repeatable methodological framework was provided by the PROSPERO database, where this review was registered in compliance with PRISMA criteria. The literature search covered two primary databases—Web of Science and PubMed—without publication timeframe limits, using a search approach integrating both controlled vocabularies and free-text terms. Search phrases were guided by discussions among the co-authors to ensure conceptual relevance.

The synthesis of findings was contextualized within international exercise guidelines for pregnancy, including those established by ACOG, RCOG, and SOGC, enabling systematic analysis of outcomes within recognized practice frameworks. This review analyzed exercise intensity levels across studies, revealing an underrepresentation of high-intensity regimens and characteristics to guide future research objectives. The subject matter corresponded with interests in physiotherapy and maternal health, potentially informing clinical issues.

Due to the linguistic restrictions of the study team, only English-language papers were included, which may have excluded high-quality research in other languages and introduced selection bias [60]. The number of studies meeting the inclusion criteria was limited, and the study methodology showed considerable variability. Most studies also had a moderate or unclear risk of bias, further limiting evidence reliability. Back pain was assessed using diverse tools, including the Visual Analog Scale (VAS), Oswestry Disability Index (ODI), Roland-Morris Disability Questionnaire, and brief interview-based measures. A systematic analysis of the pain assessment measures (e.g., VAS, ODI, RMDQ) was not conducted, as the primary aim of this research was to evaluate intervention efficacy rather than the measurement characteristics of these tools; this methodology aligns with the objective of numerous systematic reviews of interventions, which is to consolidate findings based on the metrics used in the included trials [61, 62].

Analyzed interventions varied widely in type (e.g., aerobic, water-based, strength, stability), frequency (1–7 sessions/week), duration (12–60 min/session), total length (12–36 weeks), and supervision. Inconsistent exercise intensity classification among studies posed possible bias. Because of inconsistent reporting or lack of original data, intervention intensity was deduced from narrative accounts, limiting comparability and interpretability. Most studies were conducted in Europe (67%), introducing geographical bias and limiting generalizability; this disparity illustrates uneven research production and policy focus across geographical regions. These methodological inconsistencies limited the feasibility of conducting a meta-analysis [63]. This evaluation utilized the COSMIN Risk of Bias checklist to evaluate the methodological quality of non-randomized studies, consistent with methodologies employed in prior systematic reviews [64]. None of the studies examined the biomechanical mechanisms underlying the efficacy of exercise regimens, limiting our ability to investigate how specific physical activities influence musculoskeletal functions in pregnancy-related back pain. Additionally, the included studies primarily assessed short-term outcomes and lacked long-term follow-up to evaluate the sustained effects of exercise interventions on back pain into the postpartum period. A further limitation is the potential for publication bias, which may lead to overestimation of the true efficacy of exercise interventions.

### Conclusions

This systematic review examines the impact of exercise on mitigating pregnancy-related back pain, emphasizing the effectiveness of varying interventions. Although moderate-and moderate-to-vigorous-intensity exercises have shown consistent benefits in reducing pain intensity and improving functional outcomes, the absence of high-intensity protocols such as HIIT reveals a critical research gap. Future studies are warranted to explore the safety, feasibility, and potential efficacy of high-intensity exercise in pregnancy for managing back pain.
